# Ciliated hepatic foregut cyst in a patient with renal cell carcinoma

**DOI:** 10.1186/1471-2407-6-244

**Published:** 2006-10-10

**Authors:** Todd Straus, Vladimir Osipov

**Affiliations:** 1 Department of Pathology, Medical College of Wisconsin, 9200 W. Wisconsin Ave., Milwaukee, WI53226, USA

## Abstract

**Background:**

Ciliated hepatic foregut cyst is a rare condition almost always found incidentally on a computerized tomography scan or at autopsy. Rarely, portal vein compression can be a presenting finding. The cysts are usually unilocular and occur with greater frequency in males. There is a predilection for the left lobe. The cysts average 3 cm in size.

**Case presentation:**

We present in this case report a ciliated hepatic foregut cyst found incidentally in the setting of renal carcinoma. The patient was a man known to have a large renal mass, assumed to be cancer, and a liver mass suspicious for metastatic disease. This liver mass was cystic and upon further analysis showed ciliated epithelial lining. We describe the gross and histological appearance, as well as a brief discussion of ciliated hepatic foregut cysts.

**Conclusion:**

We report the first case of a patient with coexisting renal cell carcinoma and a ciliated hepatic foregut cyst. While this may represent a coincidental finding, a possibility of a neoplastic or non-neoplastic disorder associated with ciliated hepatic foregut cysts can not be completely ruled out.

## Background

Ciliated hepatic foregut cyst (CHFC) is a rare cystic lesion of the liver found in all age groups. It was first described in 1857 by Friedrich who hypothesized its congenital derivation and was later named CHFC in 1984 by Wheeler and Edmondson [1]. Since its first descriptions less than 80 cases have been reported in the English literature. Histologically, the cyst is lined by ciliated psuedostratified columnar epithelium with scattered goblet cells. The wall of the cyst is composed of subepithelial connective tissue, 1–3 smooth muscle layers, and a fibrous outer capsule. Usually the cysts are found incidentally at autopsy or on radiology scans for unrelated conditions [2]. There are only six reported cases of CHFC in patients with cancer. The lesion rarely causes any symptoms, and when it does, usually is from mass effect on liver structures such as the portal vein [3]. For many years this lesion was considered completely benign, however there have been two reported cases of squamous cell carcinoma occurring within these cysts [4,5]. As a result, current ideas on how to manage a diagnosis of CHFC are changing. We describe the first case where CHFC was seen in a patient with renal cell carcinoma.

## Case presentation

**Figure 1:**
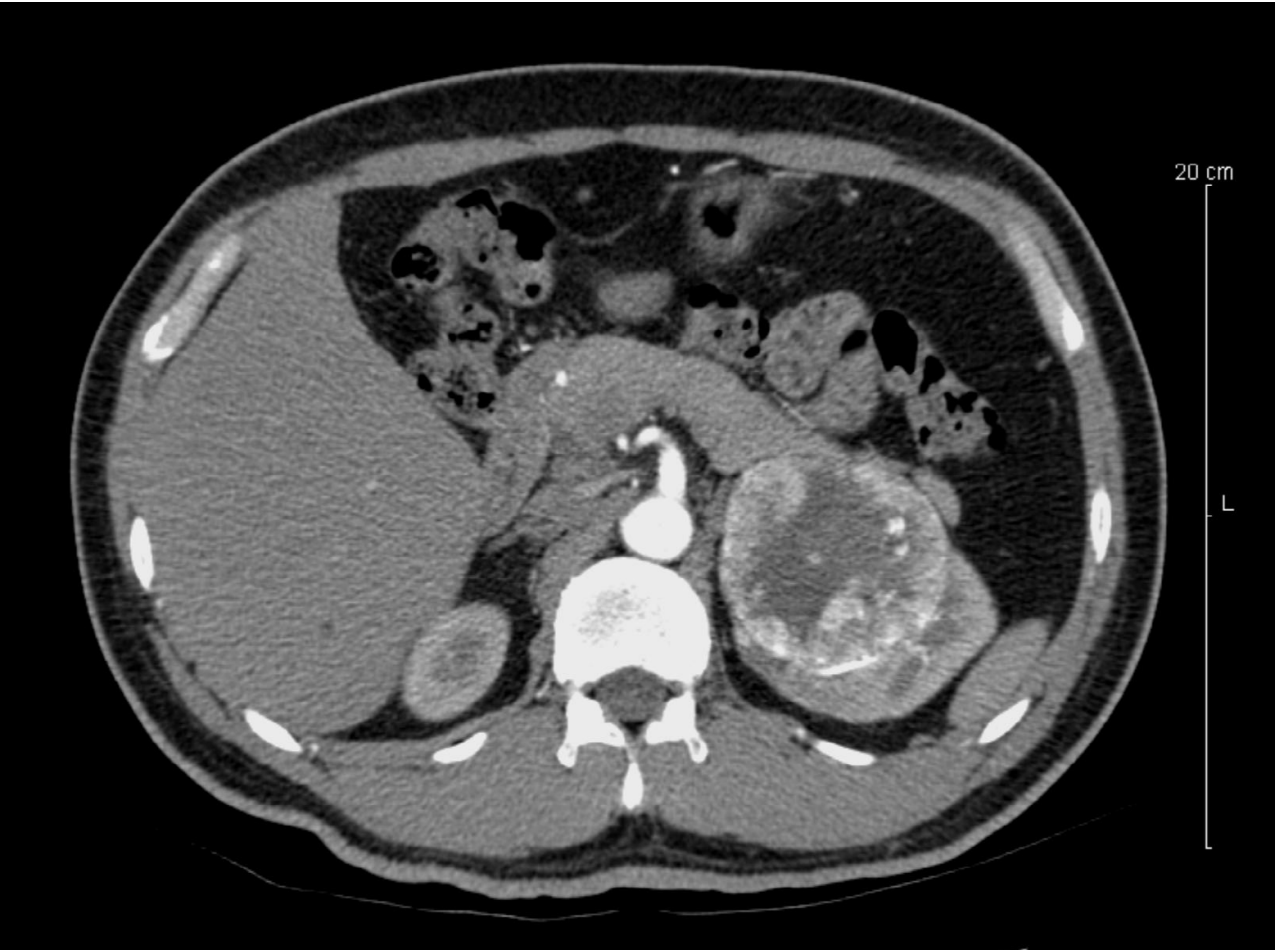
CT scan showing the renal mass.

**Figure 2:**
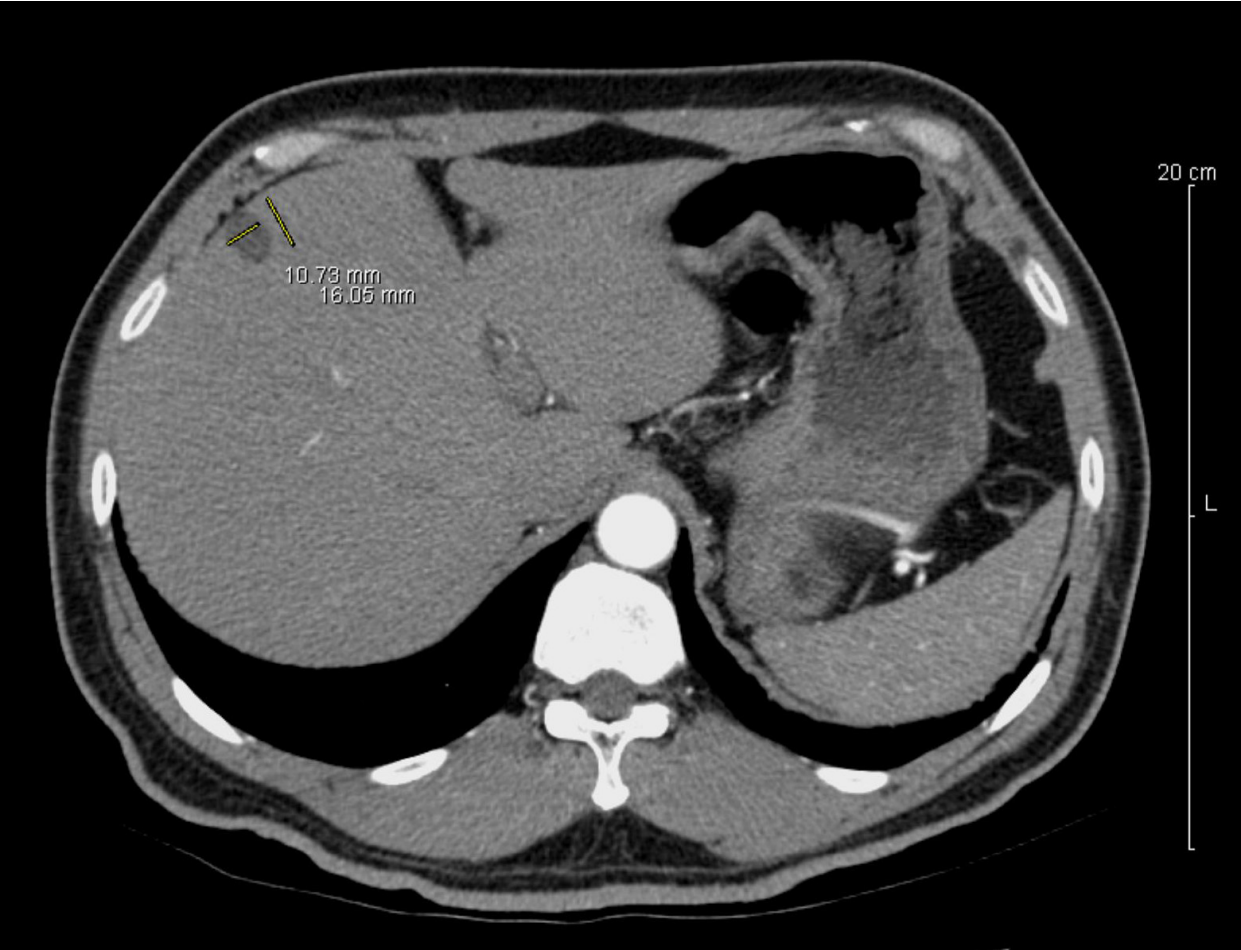
CT scan with a 1.1 × 1.6 cm hyperattenuating mass in segment 4a of the liver.

**Figure 3:**
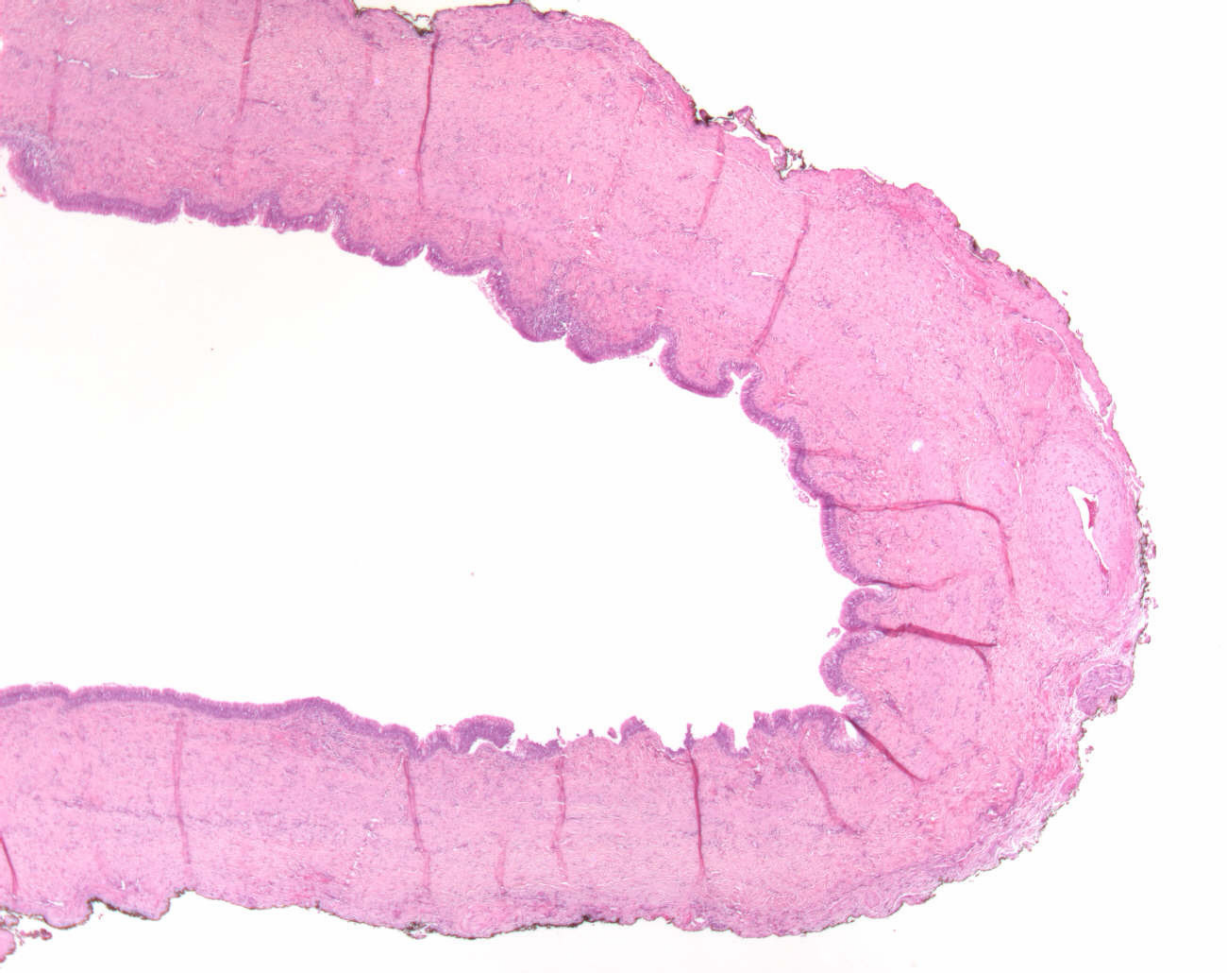
Low power image of cyst with muscular wall (hematoxylin-eosin stain × 40).

**Figure 4:**
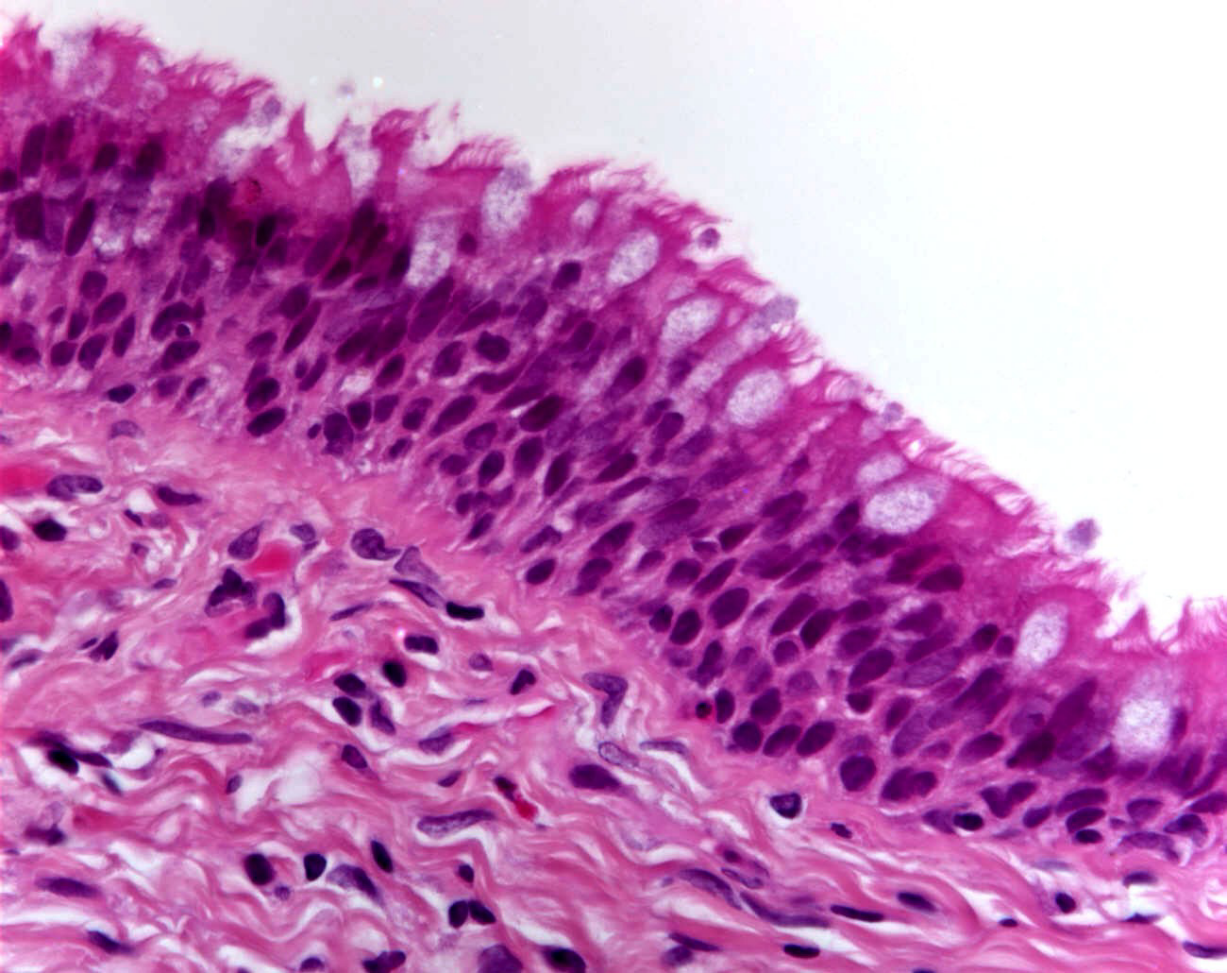
High power image of cyst lining showing ciliated columnar epithelium (hematoxylin-eosin stain × 400).

The patient is a 63-year-old man who initially presented to an outside hospital for workup of hematuria. The patient was found to have a mass in his left kidney and radiological workup had shown lesions within his liver. The patient came to our hospital seeking treatment from one of our urologists. In order to rule out a urinary bladder carcinoma, a urinary cytology was performed and showed no malignant cells. As part of his preoperative workup, the patient had CT scans of his thorax, abdomen and pelvis. The scans once again showed a large lesion on his kidney (Figure 1) and also defined multiple lesions within his liver. All but one appeared to be small simple cysts. A lesion found in segment 4a of the liver showed a circumscribed 1.1 × 1.6 cm hyperattenuating lesion pre-contrast (Figure 2) that did not enhance post-contrast. The report indicated suspicion for metastasis or hepatocellular carcinoma. Because of the concern for metastatic disease, the lesion was removed and frozen section was performed intraoperatively. A 1.5 × 1.5 × 0.5 cm tan-pink cyst was examined and diagnosed as an epithelium lined cyst. Surgery was then continued and left nephrectomy was performed for removal of the renal mass. The surgery was without complications and postoperative hospital course was uneventful. On permanent sections, the cyst was microscopically described as composed of ciliated pseudostratified columnar epithelium, subepithelial connective tissue, smooth muscle layer and outer fibrous capsule (Figure 3 and 4). A diagnosis of CHFC was given. Grossly, the renal mass consisted of a well-circumscribed nodule with a solid, golden yellow cut surface. Histologically, it was composed of malignant clear cells with a rounded to polygonal shape and abundant clear cytoplasm forming a trabecular and nesting pattern with a prominent delicate vascular network. The final diagnosis was renal cell carcinoma (RCC), clear cell type. Two years after the surgery the patient is free of disease.

## Conclusion

CHFCs are rare, mostly benign, cysts. These cysts have been reported to be 1 to 6 cm in diameter with an average of about 3 cm. The cyst in our case was of smaller size at 1.5 cm in greatest diameter. CHFCs are usually found in the left lobe of the liver, as it was in our case. Rare cases of CHFCs in the gallbladder were reported. The exact embryology of the CHFC has not yet been proven. While it is well known that ciliated cysts commonly occur in relation to the tracheobronchial tree and the esophagus, the finding of a ciliated cyst in the liver is much more rare. When the CHFC was first described by Wheeler and Edmondson[1], the histological similarities to esophageal and bronchogenic cysts were noted. This has led to a theory of development that is believed by most authors. The theory has been well written and illustrated in the paper by Chatelain et al[2]. It is generally accepted that the cysts arise from the embryonic foregut. The foregut develops into many organs in both the thorax and abdomen through a series of diverticuli and budding. It is believed that an abnormal bronchiolar bud could form and migrate down into the abdomen and be included within the liver. Because the left lobe of the liver is the dominant lobe during this time in development, this theory could explain why most CHFCs are found within the left lobe. Inclusion of an esophageal bud may also be the cause of the CHFC development. During early embryonic development the ciliated epithelium completely covers the esophagus by the 11th week and then later is replaced by mature squamous epithelium by the 24th week of gestation. This process or squamous metaplasia are likely the reasons for rare, however well-documented, cases of squamous cell carcinoma in CHFC [4,5].

## Conclusion

The CHFC is very rare and is almost always found incidentally on a CT scan or at autopsy. Review of the literature revealed that the discovery of CHFCs was made in various clinical settings, such as abdominal pain, viral hepatitis, and abnormal liver tests. In one review, 17% of CHFCs were discovered in the work-up of an extrahepatic malignancy including lung, cervical, and bladder carcinoma [2]. There have been two reported cases of malignant transformation, both of which involved invasive squamous cell carcinoma arising within a CHFC [4,5]. Although rare, the possibility of malignant transformation makes the diagnostic distinction an important one. The differential diagnosis of CHFC includes simple hepatic cyst, hepatobiliary cystadenoma and parasitic (echinococcal) cyst [6]. In the past, the treatment of choice has been aspiration or injection of the cyst with sclerosing agents, with surgical excision performed only on symptomatic lesions [7]. However, with the two more recently reported cases of malignant transformation, careful follow-up and surgical excision in most cases has been recommended [5].

We present here a case of CHFC found in the treatment of renal cell carcinoma. To the best of our knowledge, this is the first reported case of CHFC in the setting of RCC. There are total of seven (including this) reported cases when CHFCs were found in cancer patients. Three patients had lung carcinoma, one had uterine cervix carcinoma, one patient had bladder carcinoma and one had neuroendocrine carcinoma. As one can see there is much higher number of lung cancer patients which likely reflects incidence of lung cancer seen in the general population. There have not been any conditions that have been associated with CHFC to date. We could not find any possible explanation for coexistence of CHFC and renal cell carcinoma in our case either. Although it is a well known fact that hepatic cysts can be seen in association with renal cell carcinomas in the patients with Von Hipple Lindau disease, the cysts in that disease are non-ciliated. Most likely, the finding of the CHFC in this case is simply incidental. Nevertheless, until there are more cases reported, a possibility of a neoplastic or non-neoplastic disorder associated with CHFCs can not be completely ruled out.

## Abbreviations

CHFC, ciliated hepatic foregut cyst

RCC, renal cell carcinoma

## Financial competing interests

The author(s) declare that they have no competing interests.

## Authors' contributions

TS drafted the manuscript. VO reviewed and finalized the manuscript, obtained radiology and histology photographs.

## Pre-publication history

The pre-publication history for this paper can be accessed here:
